# Bibliometric Analysis of Global Research Activity on Premature Mortality

**DOI:** 10.3390/healthcare10101941

**Published:** 2022-10-03

**Authors:** Wan Shakira Rodzlan Hasani, Tengku Muhammad Hanis, Nor Asiah Muhamad, Md Asiful Islam, Chen Xin Wee, Kamarul Imran Musa

**Affiliations:** 1 Department of Community Medicine, School of Medical Sciences, Universiti Sains Malaysia, Kubang Kerian 16150, Kelantan, Malaysia; 2 Institute for Public Health, National Institutes of Health, Ministry of Health Malaysia, Setia Alam 40170, Selangor, Malaysia; 3 Sector for Evidence-Based Healthcare, National Institutes of Health, Ministry of Health Malaysia, Setia Alam 40170, Selangor, Malaysia; 4 Department of Haematology, School of Medical Sciences, Universiti Sains Malaysia, Kubang Kerian 16150, Kelantan, Malaysia; 5 WHO Collaborating Centre for Global Women’s Health, Institute of Metabolism and Systems Research, University of Birmingham, Birmingham B15 2TT, UK; 6 Department of Public Health Medicine, Faculty of Medicine, Sungai Buloh Campus, Universiti Teknologi MARA, Sungai Buloh 47000, Selangor, Malaysia

**Keywords:** premature mortality, bibliometric, research productivity, trending keywords, thematic map

## Abstract

Premature mortality is defined as death that occurs before the average age of death for a particular population. Although premature mortality is a public health problem globally, the literature indicates no bibliometric studies that have made a holistic evaluation of the publications on this issue. This study aims to explore the characteristics of the publications on premature mortality in terms of the number of publications, citations, countries, collaboration, and the author’s productivity and to further identify the trending keyword and relevant research topics. All the articles related to premature mortality data were retrieved from the Web of Science (WOS) database using the search terms “premature death,” “premature mortality,” or “years of life loss.” The retrieved articles were downloaded in a BibTeX format file. A Bibliometrix package from R software was used to perform bibliometric analyses. A total of 1060 original research articles and reviews have been published since 1971, with a total of 5499 contributing authors. The number of publications has increased substantially in the past decade. The annual percentage growth rate of publications is 5.08%. The United States is the leading country in this area of research with the highest number of publications (*n* = 280), the highest total citation (17,378), and the most activity in collaboration. Our thematic map suggests that the cluster for cardiovascular disease became the main research domain in this field, while the cluster for air pollution is an important topic for future research. Additionally, neurodegeneration is another cluster of research that should be developed further and connected with premature mortality. These bibliometric findings hopefully will help scholars better understand the global overview of premature mortality and provide information for potential collaborators, with the information promising attractive areas for future research.

## 1. Introduction

Premature mortality is the measure of unfulfilled life expectancy. According to the National Cancer Institute, premature mortality is defined as death that occurs before the average age of death in a certain population, for example, before the age of 70 [1]. It is measured in terms of Potential Years of Life Lost (PYLL) [2], the indicator for early death, where PYLL estimates the average years a person would have lived if they had not died prematurely [3]. PYLL reflects the loss of the potential contribution that younger individuals could make to society [4]. According to the National Center for Injury Prevention and Control, the WISQARS Injury data report in 2019 [5], cancer, unintentional injury, heart disease, suicide, deaths in the perinatal period, and homicide has been the leading causes of PYLL before age 75.

The trends in premature mortality globally from 1990–2017 have shown a 41% decrease in communicable diseases and neonatal disorders but a 40% increase in non-communicable diseases (NCDs) [6]. Moreover, in the United States (USA) there has been an increase in suicide and drug deaths since 2000, both contributing to the rise in premature deaths [7]. The Centers for Disease Control and Prevention (CDC) has estimated that 20–40% of premature deaths are preventable through lifestyle modifications such as smoking cessation or healthy eating and exercise [8]. In 2017, the leading global risk factors causing premature mortality for all ages worldwide were high blood pressure and smoking. However, the disease burden might be different from region to region [6]. For example, according to WHO, 85% of premature deaths due to NCDs occurred in low- and middle-income countries (LMICs) [9]. Suicide does not occur only in high-income countries. In fact, over 77% of global suicides occurred in LMICs in 2019.

Although the academic literature on premature mortality has been expanded in recent years, there is a lack of information on the total number and distribution of articles that have been published related to this area. Therefore, extracting useful information and knowledge from these existing premature mortality studies to identify current developments and future trends in this field is a high-value research topic. In this study, a bibliometric analysis approach is applied to identify the relevant publications on premature mortality that have been released globally to reach insight into this field. Bibliometrics is defined as “the application of mathematical and statistical methods to books and other media of communication” [10]. It allows for the analysis of a significant amount of scientific literature within a research area and provides a useful method to evaluate the trend in research activity over time and will help in identifying future works, novel applications, research priorities, and references within a topic. The bibliometric analysis in this study specifically aimed (1) to explore the distribution of publications in terms of the number of publications, citations, countries, collaborations, journals, and authors, (2) to identify the core journal in this field using Bradford’s law, and (3) to identify the trending keywords, co-occurrence keywords, and the most relevant clusters or themes related to premature mortality studies.

## 2. Materials and Methods

### 2.1. Data Source and Literature Inclusion Criteria

All the articles were retrieved on 29 March 2022 from the Web of Science (WOS) database. Publications were identified by searching for the terms “premature death”, “premature mortality”, “years of life loss”, or “potential years of life lost” in the title. The publications were limited to research articles and reviews in English, but no time limit was applied.

**Figure 1:**
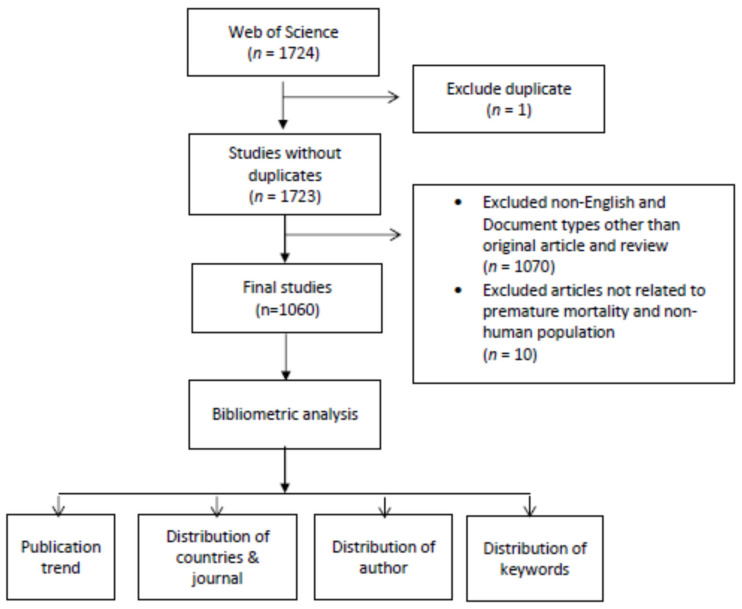
Flow diagram of the study selection process.

The publication data was downloaded in a BibTeX format from the database. The following information, or metadata, was obtained from each reference: Authors (AU), Title (TI), Publication Name (SO), Document Type (DT), Author Keywords (DE), Keywords associated by WoS (ID), Abstract (AB), Author Address (C1), Cited References (CR), Times Cited (TC), and Year (PY). After the removal of duplicates, all the publications were manually screened on the basis of titles and abstracts, and full texts if necessary. Articles that were not related to premature mortality and those that were related to the nonhuman population were excluded. Additionally, as stated previously, only research articles and review articles written in English were included. Figure 1 shows the flow diagram for study selection process.

### 2.2. Data Analysis

**Figure 2:**
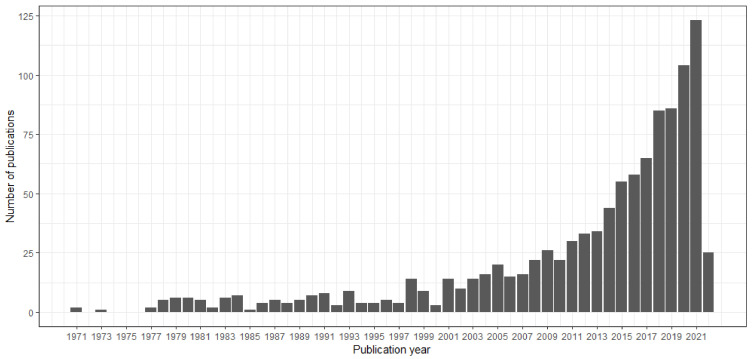
Number of articles related to premature mortality.

**Figure 3:**
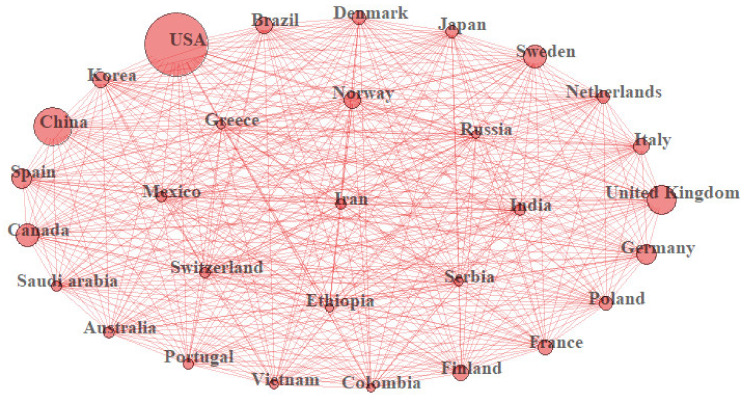
Collaboration of countries publishing articles related to premature mortality. Note: The size of the circles represents the number of collaborations with other countries.

**Figure 4:**
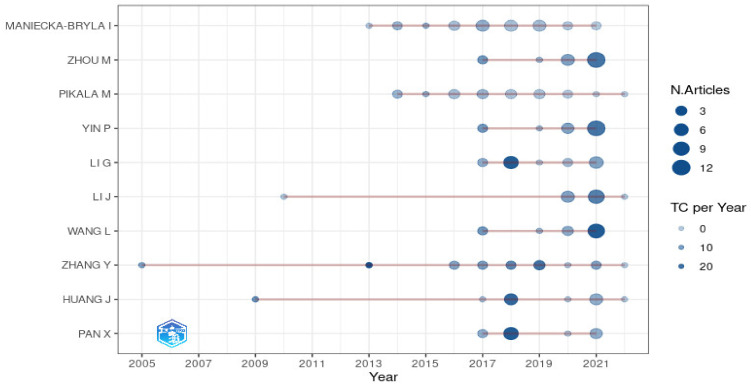
The top authors’ production over time. Note: The size of the circles represents the frequency of articles published in a year; the intensity of the circles’ color represents the papers’ relevancy.

**Figure 5:**
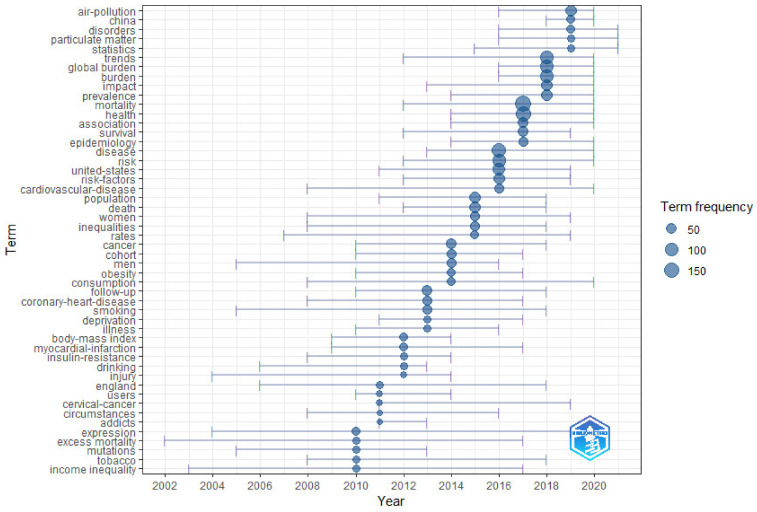
Trending keywords related to premature mortality between 2002 and 2021.

Data management and analysis were conducted using R software. To conduct bibliometric analyses, the R package Bibliometrix version 2.3.2 was used [11]. This link (https://github.com/shakirarodzlan/Bibliometric_PrematureMortality.git accessed on 1 July 2022) provides the R code for this analysis. The descriptive analysis based on the bibliometric indicators such as annual percentage growth rate (Figure 2), most productive countries (Figure 3), most productive authors (Figure 4), total citations per country, most relevant sources (journals), and most relevant keywords (Figure 5) was conducted beforehand to summarize the main results of the bibliometric analysis. In the citation analysis, we did not correct for self-citation. Moreover, for annual percentage growth rate, we calculate using this formula:

$$({\frac{Number\;of\;papers\;in\;the\;final\;year}{Number\;of\;paper\;in\;the\;first\;year}}^{1-n}-1)\times 100$$

where *n* is the number years.

The core journals in this study area were identified using Bradford’s law of scattering. The total number of publications was divided into three zones, with a relatively comparable number of publications in each zone. Collaboration analysis based on country collaboration networks was performed to demonstrate how countries relate to one another in the field of premature mortality research The number of publications according to countries was based on the countries of the corresponding authors. The collaboration index was calculated based on total authors of multi-authored articles divided by total multi-authored articles [12,13]. The collaboration index is, in other words, a co-authors-per-article index that is generated solely using the multi-authored article set.

**Figure 6:**
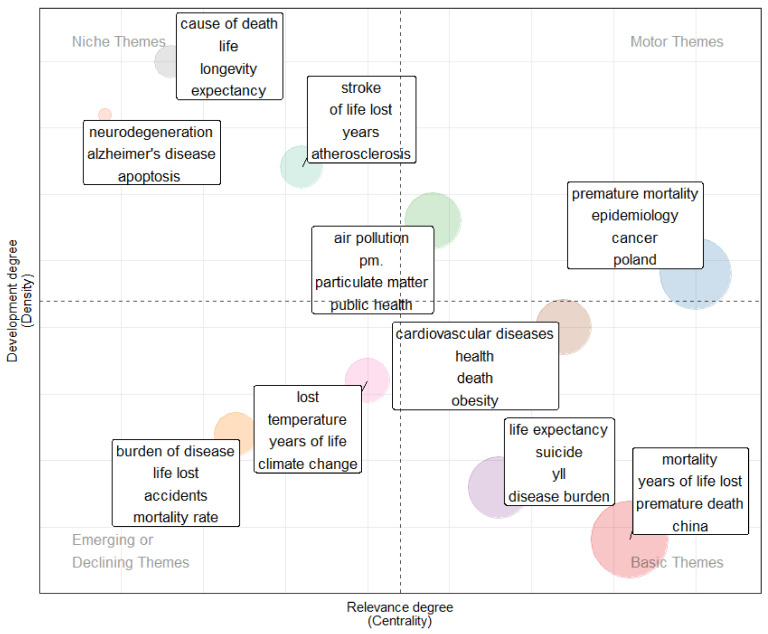
Thematic map of publications related to premature mortality.

In order to determine the themes or cluster topic, a keyword analysis was conducted. A thematic map based on the co-occurrence keyword was generated (Figure 6) on the basis of density and centrality, divided into four topological regions as proposed by Cobo et al. [14]. Density is represented in the vertical axis, and centrality takes the horizontal axis. Centrality measures the degree of correlation among different topics; density measures the cohesiveness among the nodes [15]. This map helps reveal themes that are “basic and transversal” (lower-right quadrant), “motor themes” well-developed and important for the structuring of a research field (upper-right quadrant), “emerging or declining” (lower-left quadrant), and finally, “highly developed and isolated” theme (upper-left quadrant) [14].

**Figure 7:**
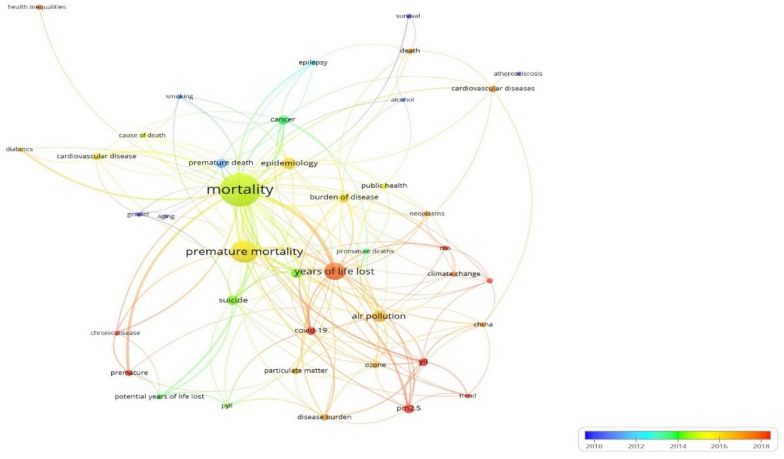
Co-occurrence keywords related to premature mortality. Note: The size of the label and the circle of an item is determined by the weight of the item. The higher the weight of the item, the larger the label and circle associated with it. The color indicates the timeline of year of publication.

Additionally, VOSviewer (version 1.6.18) software was applied to generate a network map for co-occurrence time series keywords (Figure 7). We generated a network map consisting of nodes and links based on cluster analysis. The cluster was obtained by analyzing the frequency of the same keywords appearing within the different papers. In this study, the co-occurrence of each word at least five times was considered on the basis of the title and abstract of an article. The parameter of VOSviewer for the counting method was set as full counting.

## 3. Results

The initial search returned 1724 articles related to premature mortality. After the removal of duplicates, a total of 1060 articles met the study criteria and were further included in the analysis (Figure 1). There were 1,021 original articles and 39 review articles published in the WOS database during the period 1971–March 2022. Up to March 2022, these articles were cited 44,796 times from other documents. The average citation per document and the average citation per year per document was 42.3 and 4.4 citations, respectively.

### 3.1. Publication by Year

Figure 2 shows the trend in the number of articles related to premature mortality from 1971 to 2022. The earliest article about premature mortality was published in 1971 by Gordon T., with the title “Premature mortality from coronary heart disease: The Framingham study,” and was published in the JAMA journal. From 1971 to 1997, approximately fewer than ten articles were published annually related to premature mortality. Since 1998, researchers have become more interested in this research field; this is reflected in the steady increase in the number of publications until 2010, then rapidly increasing until 2011 and peaking (123 articles) in 2021. The average year-on-year increase in publications was 5.08%.

### 3.2. Publication by Country

Table 1 lists the top 10 countries for publications related to premature mortality, listed on the basis of total publication number and total citations per country. The USA has been the top country contributing to this study area, with the highest number of publications (*n* = 269) and the highest total citations (16,308). Moreover, China is the second-highest country for publications but ranks fifth for total citations after the United Kingdom (2nd), Sweden (3rd), and Germany (4th). Figure 3 presents the bibliographic collaboration of the top 30 countries in premature mortality research. The countries with the most active collaboration have been the USA, followed by the United Kingdom and China. Most of the top countries are in Europe and other high-income areas. Other than China, there have been few representative countries of middle-income (such as Vietnam, India, Iran, Mexico, Brazil, Columbia, and Serbia) and only one country (Ethiopia) of low-income.

### 3.3. Publication by Journal

The identified articles in this study have been published in 532 journals. According to Bradford’s law, there are 32 core journals (zone 1) with a total of 354 publications. Table 2 shows the list of the top 10 core journals in order of the number of papers published. The publications are primarily science and medicine journals. The majority of the top 10 journals deal with public health. PLOS ONE has been the journal with the highest number of published articles (32), followed by the International Journal of Environmental Research and Public Health (27) and BMC Public Health (26).

### 3.4. Publication by Authors

In all, 5499 authors were involved in these articles on premature mortality. Only 77 were published as single-author documents; most were multi-authored, with a collaboration index of 5.54. The most productive authors, on the basis of the total number of publications and total citations, are presented in Table 3. Maniecka-Bryla from Poland has been the most productive author, with 26 research articles published. However, she has ranked No. 5 with respect to the total number of citations. Moreover, the analysis reveals that the top two authors with the highest total citations in this field are from China (Zhang, Y and Huang, J). Another indicator for productive authors is based on dominance factors, as proposed by Kumar & Kumar (2008) [16]. The dominance factor is a ratio indicating the fraction of multi-authored articles in which a scholar appears as the first author. In this study, Pikala M from Poland has had the highest dominance factor (0.42), where she appears as the first author in 8 out of 19 articles. This dominance factor indicates that although many researchers have been involved in relevant work, only a few authors, like Pikala M, have focused on this research area for a long time. The top 10 productive authors over time, on the basis of the number of articles and total citations per year, are presented in Figure 4.

### 3.5. Keywords Analysis

WOS records include two types of keywords: author keywords, those provided by the original authors; and keywords plus, those extracted from the titles of the cited references by Clarivate Analytics. A total of 2,418 authors’ keywords and 1,807 keywords plus terms have been discovered. Table 4 reveals the high-frequency keywords related to premature mortality based on both types of keywords. A “mortality” term has ranked first both for author keywords and keywords plus. High-frequency keywords may reflect the emerging topics in premature mortality research where most keywords are related to exposure, cause, and burden of premature mortality. Author keywords are more likely to describe specific risk factors related to premature mortality, such as air pollution, cancer, cardiovascular disease, and suicide. At the same time, keywords plus are more likely to describe broad terms (e.g., health, disease, risk, etc.) and more about methods and techniques (e.g., prevalence, survival, cohort, follow-up, etc.).

Trend topic analysis of the articles published between 2002 and 2021 shows which keywords come to the forefront over time (Figure 5). Between 2010 and 2016, the trending keywords have been focused on NCD risk factors such as tobacco, addiction, drinking, smoking, obesity, cancer, heart disease, CVD, injury, and depression. The trending topics since 2017 have been focused on the disease burden related to premature mortality, on epidemiology, and on statistics. Finally, towards 2020, the most frequent topic has been air pollution.

Figure 6 shows the thematic map of the common co-occurring keywords of premature mortality studies derived from author keywords. Ten clusters have been identified in this study, where each cluster is represented by the top four keywords. The upper right quadrant shows a motor theme, indicated by high density and centrality, consisting of the clusters “air-pollution, particulate matter, public health, and ozone”. These topics are important for future research and should be developed further. The lower-right quadrant (basic theme) is indicated by high centrality but low density. These topics are essential for research as general topics and are very important for the field’s development. They included the cluster for “mortality, year of life lost and premature death” and the cluster for “life expectancy, suicide, and disease burden”. In between the motor theme and the basic theme, we have identified a cluster for “cardiovascular disease, health, death, and obesity” and a cluster for “premature mortality with epidemiology and cancer” where these clusters have been well-developed and capable of structuring this research field. Notably from the figure, the theme of premature mortality remains the field’s leading cluster. The quadrant in the top left is indicated by high density but low centrality, including three clusters (cluster “stroke”, “cause of death”, and “neurodegeneration”). These clusters show very specialized themes, specific and under-represented topics, and rapidly developed themes. The lower-left quadrant (emerging or declining theme) is indicated by low centrality and density. In this theme, the cluster topics for “burden of disease, life lost, accident and mortality rate” have been used but have experienced a declining trend, while the cluster for “temperature, climate change, and year of life lost” has an emerging trend toward the motor theme.

Time series analysis of co-occurrence keywords was carried out using VOSviewer software to discover more about the trend topic of co-occurrence keywords. A network map of co-occurrence keywords was generated based on co-occurrence keywords greater than five, where a total of 41 keywords were selected from 1285 keywords (Figure 7). The colors of the nodes and lines represent different clusters and times. The larger the label and circle are, the greater the intensity or the number of co-occurrences between the two keywords. The thickness of the connection line represents the co-occurrence strength, which means that the thicker the connection line, the more times the two keywords co-occur. From the map, it can be seen that the co-occurrence keyword for premature death first appeared around 2010, together with risk factors for gender, age, and modifiable risk including smoking, alcohol, and atherosclerosis. At that time, survival analysis was the most frequently used keyword. Concurrent with trending keywords (Figure 5), in 2016 and onward, the term related to environmental pollution (including air pollution, particulate matter, ozone, PM 2.5, temperature, and climate change) started to emerge. Toward the current year (2022), we see “COVID-19”, a new term for co-occurrence keywords in the field of premature mortality study, appear. This link https://tinyurl.com/2ph566wj (accessed on 1 July 2022) from the VOSviewer online apps provides interactive visualization for the co-occurrence keywords in this study.

## 4. Discussion

The present study has sought to provide a detailed evaluation of the published literature on premature mortality using bibliometric indicators. Our bibliometric analysis shows that the number of publications has increased rapidly during the last decade. The huge number of publications in this research area reflects the global burden of diseases related to premature mortality. There is also quite a high number of authors (5499) participating in publishing articles related to premature mortality, and this field has received attention from researchers. This is in accordance with the World Health Organization (WHO) global target established by the United Nations Sustainable Development Goals in 2011, which aims to reduce the risk of premature death from CVD, cancer, diabetes, and chronic lung disease by one-third by the year 2030 [16]. In 2011, WHO also announced a collaboration with the journal PLOS Medicine, inviting article submissions on the theme “No health without research”, focusing on research for better health [17]. There might be a reason for the rapidly increasing number of publications since 2011. Our study also demonstrates that PLOS ONE has ranked first among the core journals publishing the most articles on premature mortality.

In terms of country distribution, it is not surprising that the USA is the leading country that has contributed to the study of premature mortality. Our findings are consistent with several bibliometric studies in other fields [18,19,20,21] which confirmed that the United States has been the global research leader in terms of the number of publications, publication quality, and collaboration participation. Other than the USA, various European countries and other high-income countries (HICs) have contributed the most to good-quality articles and have been active in collaborating in premature mortality research. Research funding, economic strength, and accessibility to research facilities are attributed to the high productivity of publications in HICs [22,23]. In this study, we have identified only one low-income country (Ethiopia) listed among the top active countries in collaboration. This demonstrates that research collaboration between low-income and HICs is still underdeveloped.

LMICs have higher relative rates of premature mortality from all three classes of disease (NCDs, injuries, and communicable diseases) than do HICs [24]. Research on premature mortality and risk factors within LMICs is critical for the development of evidence-based and context-specific policy interventions for premature mortality. However, we still lack evidence and research output about the current state of premature mortality among the poorest countries. Our findings are consistent with a previous bibliometric study which reported small numbers of authors from LMICs leading the research on poverty and NCD risk factor, and their studies are being published in relatively low-impact journals [25]. Conducting research on LMICs can be extremely challenging due to unpredictable or nonexistent research funding, weak academic institutions, and other factors including political instability. Thus, targeting funds to high-performing academics, to paucity of training, and to developing collaboration with HICs is another way of boosting impact and building research capacity in LMICs.

Our study has revealed that collaboration is the key among the authors. Only a small number of authors have published single-authored articles. The collaboration index reported at 5.56 among authors in this study is considered high. According to Lawani S.M. (1984), the collaborative index can measure the quality of research [26], and the greater the collaborative index of a set of articles, the higher the proportion of quality articles in the set. This is consistent with our finding that the USA and other HICs have been the most active countries in collaboration and, at the same time, produced the highest-quality publications with the highest total number of citations. Thus, a research collaboration among authors and countries should be encouraged.

Keyword analysis has the potential to detect current and historical trending research topics [27,28,29]. We have noticed the evaluation of keywords for premature mortality studies, especially for risk factors. Interestingly, in 2019, we detected the trending risk factor to be air pollution, whereas researchers only started to publish on this topic in 2016 and it has received little attention from then until now. Air pollution is one of the great killers nowadays, the result of rapid urbanization and deteriorating environment. According to a Global Burden of Disease (GBD) study, polluted air was responsible for 6.4 million deaths worldwide in 2015. Long-term exposure to air pollution, particularly fine particulate matter (PM2.5) and ozone, increases the risk of premature mortality [30,31]. Our thematic map suggests that the premature mortality study related to air pollution is well-developed and that the active research area is demonstrated in the motor theme (research cluster for air pollution, particulate matter, and ozone). The other research clusters for air pollution (cluster for temperature, climate change, and year of life lost) have been the emerging trend toward motor theme, suggesting this as an important topic for future research and to be developed further.

Notably, one of the research clusters from the niche theme, a cluster for neurodegeneration, has been isolated and developed internal bonds with premature mortality study, but it is still of marginal contribution to the development of this field. Neurodegenerative diseases such as Alzheimer’s disease and Parkinson’s disease are conditions that primarily affect the neurons in the human brain. This neurological disease impairs their quality of life and frequently causes them to die prematurely [32,33]. Our finding suggests that cluster research for neurodegeneration is a potential topic that needs to be more connected to premature mortality. The other important cluster for premature mortality study identified from the thematic map was cardiovascular disease. A high degree of centrality and mid-degree density of this cluster has indicated that it was recently migrated from the quadrant of a motor theme where this cluster became the main research domain related to premature mortality studies. This is an expected finding given that NCDs, particularly CVDs, have been the leading causes of premature death and continue to be a major global health challenge [34].

As another interesting finding from this study, we discovered the new emerging co-occurrence keyword for COVID-19 toward the end of our study period (2022). COVID-19 or coronavirus disease 2019 is an infectious disease caused by severe acute respiratory syndrome coronavirus 2 (SARS-CoV-2). This infection was first detected in Wuhan, China in December 2019 and became a pandemic on March 2020 [35]. Since then, publications related to COVID-19 research have been growing rapidly, including those studying the effect of COVID-19 on premature mortality [36,37,38,39]. However, as our study was conducted just a short time following the COVID-19 pandemic, we were unable to detect the true impact of COVID-19 in our bibliometric analysis. Other bibliometric studies on COVID-19 also detected the term “overall mortality” (not specific to “premature mortality” or “death”) in their keyword analysis [40,41].

Several limitations should be acknowledged. First, while focusing on the quality of journal sources, this study may relatively miss some journals that were not included in the WoS database. Secondly, for the sake of performing the keyword analysis, including a thematic map and trending keywords, this study has only included publications in English as it was impossible to perform the analysis in different languages. Third, only high-frequency keywords are selected. Thus, some important keywords with small frequencies may be ignored. Fourth, to ensure the formality and completeness of the included literature, editorials, book chapters, and conference papers are not included, which may mean that some representative publications were missed.

## 5. Conclusions

The present study summarizes the global publication information related to premature mortality, including the annual trend in publications, distribution of countries, authors’ productivity, collaborations, and keywords analysis by applying bibliometric indicators. Premature mortality research has increased substantially in the past decade, with the USA and other high-income countries dominant in premature mortality research globally. Although LMICs face higher burdens of premature mortality, primarily due to NCDs, these countries continue to have a lower-quality research output than high-income countries. Thus, research investments and collaboration are needed for LMIC to improve the development of the evidence base and reduce the burden of premature mortality. This study has also evaluated influential papers and provided information for future research regarding risk factors and the burden of premature mortality. It is hoped that the findings from this bibliometric analysis will help scholars better understand the global overview of premature mortality and provide information on potential collaborators and promising areas for future research directions.

## Figures and Tables

**Table 1:** The top 10 countries contributed to the premature mortality studies according to total publication and total citation.

	Total Publication per Country			Total Citation per Country	
No	Country	No. of Articles	%	Country	Total Citations
1	USA	269	26.3	USA	16,308
2	China	116	11.0	United Kingdom	6558
3	United Kingdom	100	9.5	Sweden	3979
4	Sweden	50	4.8	Germany	3199
5	Canada	47	4.4	China	1828
6	Australia	42	3.9	Canada	1164
7	Poland	39	3.7	Australia	1021
8	Iran	30	2.8	Switzerland	917
9	Spain	26	2.6	Denmark	911
10	Japan	26	2.4	Finland	838

**Table 2:** The core 10 journals publishing articles on premature mortality, with the number of publications.

Rank	Journal	No. of Articles
1	PLOS ONE	32
2	International Journal of Environmental Research and Public health	27
3	BMC Public Health	26
4	Science of The Total Environment	21
5	BMJ Open	19
6	Lancet	17
7	Journal of Epidemiology and Community Health	13
8	Scientific Reports	13
9	Social Science & Medicine	13
10	American Journal of Public Health	12

**Table 3:** The top 10 most productive authors of articles on premature mortality.

Rank	Authors	Total Publications	Total Citations
1	Maniecka-Bryla I	26	172
2	Yin P	20	147
3	Zhou M	20	148
4	Pikala M	19	146
5	Li G	18	190
6	Li J	16	69
7	Wang L	16	154
8	Zhang Y	15	722
9	Huang J	14	310
10	Pan X	14	178

**Table 4:** The frequent keywords in the premature mortality studies.

Rank	Author Keywords (DE)	No. of Articles	Keywords-Plus (ID)	No. of Articles
1	Mortality	213	Mortality	196
2	Premature mortality	135	Health	170
3	Year of life lost	92	Disease	138
4	Epidemiology	36	Risk	115
5	Premature death	31	Global burden	100
6	Air pollution	29	Trend	100
7	Life expectancy	29	Burden	99
8	Cancer	26	United States	80
9	Burden of disease	25	Impact	68
10	China	25	Risk factors	67
11	Suicide	25	Prevalence	63
12	Premature	24	Air-pollution	58
13	Cardiovascular disease	21	Population	55
14	PM2.5	21	Death	54
15	Particulate matter	17	Association	47
16	Potential year of life lost	17	Follow-up	47
17	YLL	17	Exposure	45
18	Cardiovascular diseases	16	Cancer	42
19	Death	16	Survival	42
20	Disease burden	16	Cohort	38

## Data Availability

All the data generated or analyzed during this study are included in this published article.
